# Nuts and Older Adults’ Health: A Narrative Review

**DOI:** 10.3390/ijerph18041848

**Published:** 2021-02-14

**Authors:** Sze-Yen Tan, Siew Ling Tey, Rachel Brown

**Affiliations:** 1 Institute for Physical Activity and Nutrition (IPAN), School of Exercise and Nutrition Sciences, Deakin University, Geelong, VIC 3220, Australia; 2 Department of Human Nutrition, University of Otago, P.O. Box 56, Dunedin 9054, New Zealand; siewling.tey@otago.ac.nz (S.L.T.); rachel.brown@otago.ac.nz (R.B.)

**Keywords:** nuts, older adults, ageing, quality of life, telomere, sarcopenia, cognition, diet quality

## Abstract

Although the beneficial effects of nuts on cardiometabolic diseases have been well established, little is known about the effects of nuts on age-related diseases. Given that age-related diseases share many biological pathways with cardiometabolic diseases, it is plausible that diets rich in nuts might be beneficial in ameliorating age-related conditions. The objective of this review was to summarise the findings from studies that have examined the associations or effects of nut consumption, either alone or as part of the dietary pattern, on three major age-related factors—telomere length, sarcopenia, and cognitive function—in older adults. Overall, the currently available evidence suggests that nut consumption, particularly when consumed as part of a healthy diet or over a prolonged period, is associated with positive outcomes such as longer telomere length, reduced risk of sarcopenia, and better cognition in older adults. Future studies that are interventional, long-term, and adequately powered are required to draw definitive conclusions on the effects of nut consumption on age-related diseases, in order to inform dietary recommendations to incorporate nuts into the habitual diet of older adults.

## 1. Introduction

Human life expectancy has increased globally, and the increment rate has been more rapid in industrialised countries [1]. Greater life expectancy can be largely attributed to medical advancements, which significantly reduces mortality rates [2]. Due to increased longevity, healthy ageing and better quality of life are becoming more important among older adults [3]. The quality of life of older adults is multidimensional and depends on: (a) individual factors, e.g., satisfaction with one own’s physical/mental health, functional capacity (autonomy), emotional comfort, spirituality, and financial security; and (b) environmental factors, e.g., social interaction, network, and support [4].

**Figure 1:**
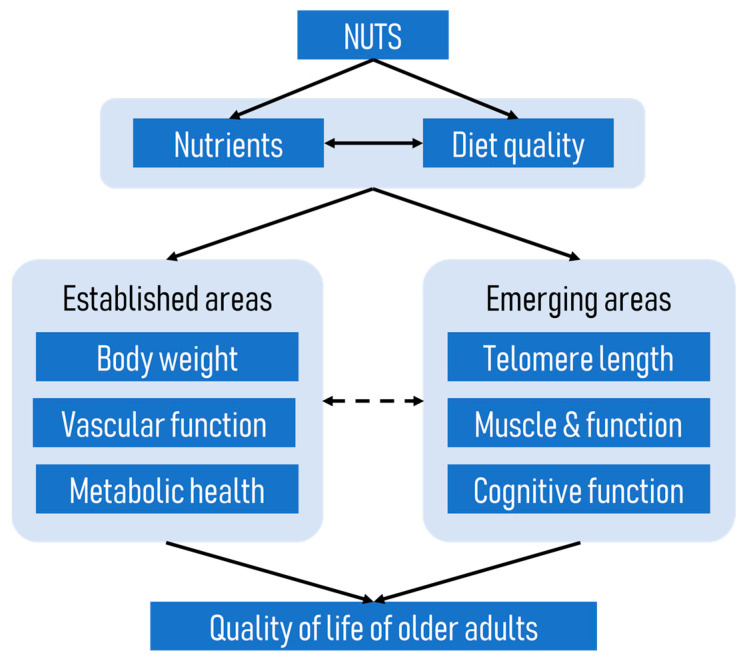
A conceptual framework of how nuts improve the quality of life of older adults.

The focus of this review is on how nut intake either consumed alone or as part of the dietary pattern can improve the quality of life of older adults. We propose that nuts may improve the quality of life of older adults through the promotion of better health, cognitive function, and functional capacity in this population, as depicted in a conceptual framework below (Figure 1). This framework is based on the premise that nuts, which are high in essential nutrients, improve diet quality and the overall nutritional status of older adults (see previous review [5]). As outlined in this framework, better nutrition and diet quality will, in turn, improve the health, wellbeing, and the quality of life of older adults.

A number of previous reviews have highlighted the benefits of nuts on body weight regulation [6,7], improved vascular function, and prevention of cancer [8] and metabolic diseases such as cardiovascular disease [9,10] and type 2 diabetes mellitus [10,11], which are prevalent among older individuals; therefore, these aspects will not be addressed again in this review. Instead, we will explore other emerging areas such as the potential effects of nuts on telomere length, muscle and function, and cognitive function of older adults. These aspects are of importance because telomere length has been shown to be an important indicator of ageing [12], while optimal physical and cognitive function will allow older adults to live independently as long as possible. It is also important to note that the emerging areas discussed in this review are not independent of the well-established areas shown in Figure 1. For example, telomere length has been linked to the onset of several age-related diseases [13], and vascular function has been shown to influence cognitive function [14].

In this narrative review, we summarise the findings from studies that have investigated the associations or effects of nut consumption on telomere length, muscle and function, and cognitive function of older adults. These are emerging areas in nut research; therefore, we have included studies that have examined the influence of nuts alone, or nuts as part of an overall dietary pattern such as the Mediterranean diet. In the latter, it is often difficult to attribute the observations to nuts or the nuts/seeds/legumes food group within the dietary patterns specifically, unless these studies conducted separate analysis on each of the components within the dietary patterns. Hence, the results from studies that focused primarily on dietary patterns only should not be over-interpreted. Furthermore, although different nuts are high in certain nutrients, all nuts share very similar overall nutritional profiles, i.e., high in unsaturated fats, fibre, and nutrients that are essential for good health [5]. For this reason, all nut types are considered comparable nutritionally, and dietary recommendations focus on all nuts instead of specific nut types. Therefore, this review will not compare different nut types, but rather consider them collectively. Finally, studies cited in this review were not limited to those that included older adults (>60 years) only, but they were included nonetheless because optimal nutrition in younger adults is arguably very important in the prevention of health conditions that occur in old age.

## 2. Nut Consumption and Telomere Length

Regular nut consumption is associated with a reduced risk of chronic diseases [15,16], including biomarkers of these age-related diseases [10,17,18]. The effects of nut consumption on telomere length have gained attention as one possible mechanism whereby nuts may reduce age-related diseases [19]. Telomeres are caps which protect the ends of chromosomes, and their lengths are an indicator of biological age. They protect DNA from oxidative damage, allowing the cell to divide normally. The length of telomeres is carefully controlled by a variety of proteins, including the enzyme telomerase, which promotes telomere length and stability [20]. Telomere length is shortened with each cell division, with a loss of around 50–200 bases [21]. Eventually, the telomere will reach a length that is associated with cell apoptosis [22]. Shortening of telomeres is negatively associated with cell longevity, and has been associated with disorders such as cancer, cardiovascular disease, neurodegenerative diseases, hypertension, and type 2 diabetes [23,24].

Although increasing age is strongly correlated with shorter telomere length, the variability in the rate of telomere shortening—independent of chronological age—suggests that other factors are important. Several modifiable lifestyle factors have been implicated in the rate of telomere shortening. For example, telomere length has been positively associated with greater fruit and vegetable intakes, and higher levels of physical activity; and negatively associated with higher saturated fat and meat consumption intakes, adiposity, and smoking [25,26,27,28,29,30]. The following sections review the evidence for the association of nut consumption and telomere length to determine whether this may partly explain the reduction in age-related diseases observed with regular nut intake. 

There are several potential mechanisms whereby nuts may exert a positive effect on telomere length and cellular senescence. A number of nutrients have been implicated as having an important role in DNA methylation and integrity due to their antioxidant properties [31]. These nutrients include isoflavanoids, folate, vitamin E, and polyunsaturated fatty acids (PUFAs). Although nuts differ in a number of individual nutrients, they are all rich sources of antioxidant nutrients and unsaturated fatty acids. For example, peanuts and hazelnuts are particularly good sources of folate, whereas walnuts, Brazil nuts, and pine nuts are good sources of PUFA. It has been suggested that telomere length is a marker of oxidative stress [32]. Previous studies have shown that regular nut consumption is associated with reductions in some, but not all, markers of oxidative stress and inflammation [33,34]. In addition, Cannudas et al. [35] showed reduced oxidative damage of DNA with the consumption of pistachio nuts over four months.

To examine the association between nut consumption and telomere length, we reviewed one prospective study and two intervention studies that specifically examined the independent effect of nut consumption on telomere length. We also reviewed fifteen cross-sectional analyses, two prospective analyses, and two intervention studies that included or emphasised nuts as part of a dietary pattern. 

### 2.1. Evidence from Observational Studies

#### 2.1.1. Nut-Specific Studies

One study from a large nationally representative sample examined the association between nut consumption and telomere length [19]. Tucker et al., using 24 h recall data from the National Health and Nutrition Examination Survey (NHANES) 1999–2002 (*n* = 5582), found a positive association between the consumption of nuts and seeds and telomere length. The association was linear (after adjustment), with each 1% of total energy derived from nuts and seeds associated with a length which was 4.5 base pairs longer. To give some perspective to these results, the authors calculated that using an estimated age-related rate of shortening of telomeres of 15.4 base pairs per year, adults of the same age who consumed 5% of total energy from nuts and seeds had around one- to two-thirds less cell ageing compared to non-consumers.

#### 2.1.2. Studies on Dietary Patterns That Include Nuts

Observational studies that have examined the association between telomere length and diet quality or dietary patterns that include or emphasise nut consumption have been performed in a range of different ethnic groups and countries, including Korea [27], China [36,37], Spain [38,39], Hong Kong [40], Italy [41], the United States [25,42,43,44], Iran [45], Australia [46], and Finland [47]. Of the fifteen observational studies, thirteen used a cross-sectional design [25,36,37,38,39,40,41,42,43,44,45,46], one study [27] used a prospective design, and one study included both a cross-sectional and prospective analysis [47]. Details of these studies can be found in Table 1. Although the dietary indices used in these studies do not allow us to identify the independent effects of nuts, nuts were a food group which comprised the healthy component of these indices.

Nine studies examined adherence to pre-determined diet quality indices which emphasised nut intake. These included the Mediterranean diet score, Dietary Approaches to Stop Hypertension (DASH) score, Health Eating Index 2010 score (HEI-2010), Alternative Health Eating Index 2010 score (AHEI-2010), and the Prime Diet Quality Score (PDQS). 

##### Studies Using a Mediterranean Diet Quality Score

The dietary pattern which has received the most attention in this area is the Mediterranean diet, which has been correlated with healthy ageing and longevity [48]. One of the key components of the Mediterranean dietary pattern is nut consumption. In addition, this pattern is characterised as being largely plant-based with high amounts of olive oil, fruits and vegetables, legumes, and wholegrains [49]. When study populations were analysed as a whole, three studies showed a positive association between the Mediterranean diet and telomere length [25,39,41], while two showed no association [44,46]. Two studies showed positive associations in women only [38,43], and one U.S. study showed a positive association among white, but not African American or Hispanic participants [42]. Meinilä et al. found no association in a cross-sectional analysis of their Finnish population, but found slight, although statistically significantly, higher rates of telomere shortening among women adhering to a Mediterranean dietary pattern in the 10-year follow-up prospective analysis. Interestingly, this was largely driven by the fruit and nut food group; however, this difference was small and not considered clinically important [47]. 

Two studies that showed a positive association with the Mediterranean dietary pattern also analysed nuts as a food group, but found no association between nuts and telomere length [25,38]. Trichopoulou et al. have previously suggested that components of the Mediterranean eating pattern may be additive; hence, the lack of association based on a single nut food group. Additionally, looking at individual foods may be more susceptible to residual confounding [50]. Therefore, the potential benefit of nuts on telomere length is likely to be mediated through better overall diet quality. Of note is the finding that studies on the Mediterranean eating pattern carried out in southern European countries tended to show positive associations with telomere length compared to those carried out in countries such as Australia [46], Finland [47], and the United States [44]. This may indicate higher overall adherence to such an eating pattern in southern Europe, which may be more likely to show positive associations.

A meta-analysis including eight of the aforementioned cross-sectional studies collectively assessed the association between adherence to the Mediterranean diet and telomere length maintenance [51]. In the fully adjusted model for all participants, there was a positive association between adherence to the Mediterranean diet and telomere length maintenance, but this association disappeared when males and females were separated. This may be due to reduced power, but of note, no significant associations were seen in any of the models for men.

##### Studies Using Other Diet Quality Scores

Two of the aforementioned studies that investigated the Mediterranean diet also examined other nut-containing dietary patterns [39,43]. Ojeda-Rodriquez showed that, similar to their findings of the Mediterranean diet, greater adherence to the Prime Diet Quality Score (PDQS), the Alternative Healthy Eating Index 2010 score (AHEI-2010), and Dietary Approaches to Stop Hypertension (DASH) scores was associated with longer telomere length among the Seguimiento Univeridad de Navarra (SUN) cohort [39]. In contrast, Leung et al., using data from the 1999–2002 cycles of the National Health and Nutrition Examinations Survey (NHANES), showed significant trends for longer telomere lengths from the lowest to the highest quintiles for each diet score (the Healthy Eating Index 2010 score (HEI-2010), the Alternative Healthy Eating Index 2010 score (AHEI-2010), or the DASH diet score) among women, but not men. This reflects their findings in terms of adherence to the Mediterranean diet [43].

##### Posteriori Dietary Patterns 

Three studies (two cross-sectional and one prospective) analysed FFQ data and derived dietary patterns using principal component analysis (PCA) or factor analysis [27,36,52]. One study conducted in South China found that a dietary pattern containing nuts was associated with longer telomeres among women, but not men [36]. In a prospective study conducted in South Korea, a prudent diet was positively associated with telomere length [27]. When analysing individual food items which contributed to the prudent dietary pattern, nuts were positively associated with telomere length. 

One U.S. study, including data from 840 Black, White, and Hispanic adults taking part in the Multi-Ethnic Study of Atherosclerosis (MESA), used PCA and reported no association between consuming a nut-containing food pattern and telomere length [52]. They also failed to show an association when nut or seeds were considered alone. 

##### Studies on Food Group Including Nuts

A further three studies used FFQs to examine the association of nuts and seeds consumed as a food group and telomere length. One study conducted in China showed that intakes of nuts or seeds were highest among those in the upper tertile for telomere length, and intakes were positively associated with telomere length [37]. In contrast, Chan et al., reported no associations between nut consumption and telomere length in a cross-sectional study of 2006 Chinese males and females living in Hong Kong [40]. Here, nuts were combined with the food group of legumes, seeds and nuts. A further study among males in Iran showed that nuts and seed were negatively, albeit not statistically significantly, associated with telomere length [45]. It should be noted that participants in this study were younger (25–40 years) compared to most other studies in this area.

#### 2.1.3. Summary of Studies on Dietary Quality and Patterns

Collectively, these observational studies on dietary patterns containing nuts have produced mixed results, making it difficult to form conclusions. Of the fifteen studies, nine showed a positive association between the consumption of a nut-containing dietary pattern and telomere length in the population as a whole or in a sub-group, five showed no association, and one showed a negative association, albeit not clinically important. Some factors which make interpretation of these observational studies difficult include the inability to examine the independent effects of nuts, variation in the age of participants, and the use of different dietary assessment tools. There also appears to be some sex differences, with three studies showing positive associations with consumption of nut-containing dietary patterns and telomere length in females only [36,38,43]. The reason for these sex-specific associations cannot be speculated based on the observational nature of these studies, and future clinical trials are therefore needed to understand the potential underlying biological explanations. A further factor to consider when interpreting the results of observational studies is survivor bias. This is where people who live longer tend to be more resilient to chronic disease. Although these studies cannot infer causality, they do provide useful information on which to base hypotheses that can be explored in intervention studies.

### 2.2. Evidence from Interventional Studies

#### 2.2.1. Nut-Specific Studies

To the best of our knowledge, only two intervention studies have specifically assessed the effects of nut consumption on telomere length [35,53]. Freiras-Simoes et al. examined the inclusion of 15% of dietary energy from walnuts (*n* = 80), compared to a control group (*n* = 69), who continued to consume their usual diet while abstaining from walnuts, on the maintenance of telomere length in a group aged 63–79 years [53]. This parallel study was conducted over two years. There was a significant increase in red blood cell alpha-linolenic acid in the walnut group compared to the control, indicating compliance to the interventions. There was a tendency (*p* = 0.079) for the control group to have greater reduction in telomere length over the two years compared to the walnut group. In addition, the change in the percentage of telomeres with lengths less than 3 kb at the end of two years was marginally statistically significantly lower in the walnut group. Taken together, these results suggest that consuming walnuts may reduce telomere attrition.

Canudas et al. investigated the effect of pistachio consumption on telomere length and gene expression related to telomere maintenance in 49 participants aged 25–65 years with pre-diabetes using a crossover design [35]. Participants consumed a diet supplemented with 57 g/d pistachios compared with an isocaloric control diet for four months each. Telomere length did not differ between the two treatments; however, genes associated with telomere length were significantly upregulated in the pistachio treatment compared with the control. 

While the findings of these intervention studies are promising, they need to be confirmed in long-term, adequately powered, randomised intervention studies using different types and doses of nuts.

#### 2.2.2. Studies on Dietary Patterns That Include Nuts

There are only two intervention studies which have assessed telomere length in response to interventions with dietary patterns that emphasise nut consumption. 

Garcia-Calzón et al. performed several analyses on the association between telomere length and diet using a subgroup from the PREDIMED-NAVARRA trial involving 520 participants aged 55–80 years who were at high risk of CVD [38]. In this trial, participants were randomly assigned to one of two Mediterranean diets supplemented with either extra virgin olive oil or mixed nuts, or to a low-fat control diet. Intervention with the Mediterranean diets with nuts was associated with a higher risk of telomere shortening compared to the control, whereas, there were no differences between the Mediterranean diet with extra virgin olive oil and the control group [38]. When analysing the individual components of the Mediterranean diet including nuts and extra virgin olive oil, there was no association between these components and telomere length. This is in agreement with other observational studies [25,42]. The sample size may not have had sufficient power to identify small changes in telomere length by individual dietary components. The finding that the intervention with the Mediterranean diet with nuts had a detrimental effect on telomere length was unexpected; although, it should also be noted that the control group increased adherence to the Mediterranean diet over the intervention, which made the results difficult to interpret. The authors also suggest that lifetime exposure to a Mediterranean diet may be more meaningful in determining telomere length than exposure during a five-year intervention. Further analysis of this group showed that among those with the Pro12Ala polymorphism—a gene variant associated with lower CVD risk—greater adherence to the Mediterranean dietary pattern was associated with greater prevention of telomere shortening [54]. 

In a small crossover study, 20 participants aged over 65 years consumed three diets for four weeks each: a Mediterranean diet enriched in olive oil; a saturated fat-rich diet; and a low fat, high carbohydrate diet enriched in *n*-3 PUFA from walnuts [28]. The Mediterranean diet was associated with a lower percentage of telomere shortening compared to the other two interventions. It was proposed that the Mediterranean diet protected against oxidative stress, and thus prevented telomere shortening. A Mediterranean diet usually contains nuts; however, it is unclear to what extent nuts were included in the Mediterranean arm of this study. 

### 2.3. Summary 

Overall, research on the effects of nut consumption and telomere length is inconsistent. Some observational studies show greater telomere length among those consuming dietary patterns including nuts, while others do not. Many of these studies failed to show an association with nuts consumption per se. Several systematic reviews have shown that intensive lifestyle interventions delay telomere shortening [29]. Therefore, more comprehensive changes may be more effective than changing only one component of the diet, such as increasing nut consumption. This suggests that the synergistic effect of nutrients may be important. It is possible that nuts, as part of a healthy diet and lifestyle, may be one contributing factor telomere health, and may be one of the underlying mechanisms whereby regular nut consumption reduces the risk of age-related diseases. However, it is important to note that much evidence derives from observational studies, and hence the relationship between nut intake and telomere length is largely associative rather than causal. How telomere length translates into lifespan is not straightforward, so it is also not possible to propose that nut intake contributes to longevity in older adults.

## 3. Nut Consumption and Sarcopenia and Related Factors

According to the revised European Working Group on Sarcopenia in Older People 2 (EWGSOP2), sarcopenia is characterised by low levels of muscle strength and muscle quantity or quality, and severe sarcopenia is characterised by sarcopenia and low physical performance [55]. 

Ageing is associated with the loss of muscle mass and strength, although the rate of decline differs between individuals, suggesting that lifestyle factors such as diet and physical activity may be important determinants of muscle health [56,57]. Current evidence suggests that nutrients such as protein, beta-hydroxy-beta-methylbutyrate, vitamin D, antioxidant nutrients, and long-chain PUFAs, as well as physical activity, may ameliorate the risk of sarcopenia [57,58,59], through muscle protein synthesis or preventing muscle breakdown, which helps to preserve muscle mass and function. Nuts are rich sources of plant protein, unsaturated fatty acids, phytochemicals, vitamins and minerals; therefore, these nutrients may act synergistically for the prevention and management of sarcopenia in older adults. 

This section will review observational studies that examined the association between nut consumption, either alone (Section 3.1.1) or as part of a dietary pattern (Section 3.1.2), and sarcopenia and related factors in older adults. A total of seven observational studies have been identified (Table 2) [60,61,62,63,64,65,66]. To the best of our knowledge, no intervention studies have been specifically designed to determine the effect of nut consumption on sarcopenia.

### 3.1. Evidence from Observational Studies

Seven observational studies have been identified in the nuts and sarcopenia area (Table 2) [60,61,62,63,64,65,66]. One prospective study examined the association between nut consumption and physical function [60], three observational studies (one prospective study and two cross-sectional studies) reported nuts as a food group [62,63,65], and another three studies (one prospective study and two cross-sectional studies) examined the adherence to diet quality indices in which nuts was a key component [61,64,66]. Two studies were conducted in Spain, and one each in the United States, China, Korea, Denmark, and Iran (Table 2). 

#### 3.1.1. Nut-Specific Studies

To date, only one prospective study has examined the association between nut consumption and physical function [60]. The Seniors-ENRICA cohort study was conducted in Spain in 3289 community-dwelling older adults aged ≥60 years [60]. A validated diet history was used to assess consumption of 20 types of nuts in this study. Physical function was ascertained by five domains, namely agility, mobility, overall physical function, grip strength, and gait speed, with the first three domains being self-reported and the last two domains being objective measures.

Participants were classified into three categories based on their nut consumption: non-consumers, <median (<11.5 g/d), and ≥median (≥11.5 g/d). In men, there was a dose-response relationship between nut consumption and impaired agility (*p* for trend = 0.01) and mobility (*p* for trend = 0.02), in which higher nut consumption was associated with lower risk of these two impairments. Compared with non-consumers, nut intake of ≥11.5 g/d was associated with a lower risk of impaired agility (HR = 0.59, 95% CI: 0.39, 0.90) and impaired mobility (HR = 0.50, 95% CI: 0.28, 0.90) in the fully adjusted models. In women, there was a dose-response relationship between nut consumption and impaired overall physical function (*p* for trend = 0.004). Compared with non-consumers, nut intake ≥11.5 g/d was associated with lower risk of impaired overall physical function (HR = 0.65, 95% CI: 0.48, 0.87). However, nut consumption was not associated with grip strength and gait speed. The authors reported that this could be due to the low sensitivity of method used to detect the differences.

Overall, this study revealed that nut consumption was associated with a lower risk of self-reported impaired agility and mobility in men, and lower risk of impaired overall physical function in women [60]. However, such associations were not observed in objective measures of grip strength and gait speed. Further investigation is warranted to confirm this finding.

#### 3.1.2. Studies on Dietary Patterns That Include Nuts

Six observational studies have examined the association between dietary patterns that include nuts and sarcopenia. Three studies reported nuts as a food group [62,63,65], while the other three studies reported the adherence to diet quality indices in which nuts was a key component [61,64,66].

##### Studies on Dietary Patterns That Include Nuts (as a Food Group)

Three studies, consisting of one prospective study and two cross-sectional studies, examined nuts as part of a dietary pattern and their relationship with muscle mass and function [62] and sarcopenia [63,65]. The Framingham Offspring study was the first prospective study to investigate the association between a diet rich in protein-source foods (including nuts) and skeletal muscle mass and functional status among community-dwelling adults [62]. Results showed that compared to consumption <0.25 servings/day of “legumes, soy, nuts, seeds”, those who consumed ≥1.25 servings/day had a higher mean percent skeletal muscle mass in men (difference of 0.7%, *p* = 0.0197) and women (difference of 0.8%, *p* = 0.0156), although the difference in percent skeletal muscle mass was small.

Two cross-sectional studies in China and Korea examined the association between food groups (in which nuts was one of the food groups) and sarcopenia in older adults [63,65]. Hai et al. [63] reported that female participants with sarcopenia had a significantly lower frequency of nut consumption compared to those without sarcopenia (0.05 times per week vs. 0.81 times per week, *p* = 0.022). In addition, there was a 28% reduction in the prevalence of sarcopenia and frequency of nut consumption (OR = 0.724, 95% CI: 0.532, 0.985). In line with these findings, a cross-sectional study showed that male participants with sarcopenia had a significantly lower intake of nuts and seeds compared to those without sarcopenia (3.1 g per day vs. 5.2 g per day, *p* = 0.002) [65]. 

Overall, the evidence from observational studies where nuts were included as a food group showed an inverse association between nut consumption and sarcopenia [63,65]. In addition, a prospective study reported higher skeletal muscle mass among people who consumed more servings/day of “legumes, soy, nuts, seeds” [62]. The current, albeit limited, epidemiologic evidence suggests a protective effect of nut consumption on sarcopenia in older adults. 

##### Studies Using Diet Quality Indices

Three studies, including one prospective study and two cross-sectional studies, examined adherence to diet quality indices, such as the Mediterranean diet and Mobility diet, and the risk of sarcopenia and related factors [61,64,66]. The Seniors-ENRICA prospective study reported a dose-response relationship between the Mediterranean Diet Adherence Screener (MEDAS) score and risk of falling (*p* for trend = 0.04) [61]. Participants in the highest tertile of the MEDAS score had a 28% reduction in the risk of falling, in comparison with those in the lowest tertile of the MEDAS score. Similarly, Hashemi et al. [64] reported an inverse dose-response relationship between the Mediterranean pattern and sarcopenia (*p* for trend = 0.04). Participants in the highest tertile of the Mediterranean pattern had a 60% reduction in the odds of having sarcopenia, compared to those in the lowest tertile of the Mediterranean pattern. There was a significant association between a Mediterranean dietary pattern and prevalence of low gait speed (*p* = 0.02). The percentage of participants with low gait speed (<0.8 m/s) was lowest in the top tertile of the Mediterranean dietary pattern. In line with these findings, another cross-sectional study reported that dietary index characterised by higher intakes of whole grains, dairy products, fish, legumes, nuts, fruit, and vegetables was associated with faster 400 m walking speed (*p* for trend = 0.021) [66].

Overall, the evidence showed an association between dietary patterns with nuts and a lower risk of falling, sarcopenia, and low gait speed. Nuts were one of the many components in these diet quality indices, which makes it difficult to estimate the independent effect of nuts. It is likely that the combined effect of several food components within a diet will exert greater protective benefits than the individual effect of a single food. Nevertheless, these results are promising and warrant further investigation.

### 3.2. Summary 

There is limited published research investigating the association between nut consumption and sarcopenia and its components. Results from the prospective studies and cross-sectional studies reported positive associations between nut consumption and physical function. It is important to note that no study to date has reported a detrimental effect on muscle function after nut consumption. Further research is required to draw definitive conclusions of the association between nut consumption and sarcopenia.

## 4. Nut Consumption and Cognitive Function 

The potential benefits of nuts on cognitive function have been proposed in a previous review, which included three observational studies and one interventional trial [14]. The authors summarised that nut consumption appears to be associated with better cognition, hypothesising that this relationship may be explained by improved endothelial function, which subsequently improves cerebral blood flow and the delivery of nutrients with anti-inflammatory properties to the brain. The studies included in the review by Barbour and colleagues did not focus on older adults specifically [67,68], but also included young [69] and middle-aged adults [70]. Therefore, for the purpose of this paper, we conducted a literature review of studies that were published since the previous review, which explicitly focused on older adults. In total, we identified 18 studies (12 observational and 6 interventional studies) that reported on nut consumption and cognitive function of older adults (Table 3 and Table 4). 

### 4.1. Evidence from Observational Studies

#### 4.1.1. Nut-Specific Studies

Six studies (three cross-sectional and three prospective) investigated the relationship between cognitive function of older adults with nut intake specifically (Table 3). Only one study did not find significant differences in the cognitive battery test scores between older adults who have low vs. high nut intake after adjusting for multiple covariates [67]. The remaining five studies reported significant and positive associations between various measures of cognitive function and nut consumption of older adults [68,71,72,73,74]. Specifically, higher nut consumption was related to better overall cognition [72,73], working memory [68,73], and immediate recall [73]. In prospective observational studies, nut consumption was also associated with a lower probability (OR: 0.78, 95% CI: 0.61–0.99) of cognitive decline over three years [74], and participants were 40% less likely to have poor cognitive function [72]. However, the association between nut consumption and slower cognitive decline was not observed in another study that only included women aged 70 years and over [73].

#### 4.1.2. Studies on Dietary Patterns That Include Nuts

Six studies (three cross-sectional and three prospective) also investigated the associations between dietary patterns that included nuts and cognitive function of older adults. In these studies, nuts were either a stand-alone component of a dietary pattern [75,76,77,78], or as part of a bigger food group such as the “nuts and legumes” [79] or “pulses, nuts and seeds” food groups [80]. Therefore, it is important to note that it was not always possible to attribute the findings of these studies to the intake of nuts specifically. Again, only one study (with approximately a nine-year follow-up period) did not find significant associations between nut consumption and global cognition or verbal memory of over 6000 older women [77]. In the remaining five studies, three cross-sectional studies reported a lower risk of cognitive impairment with a higher intake of nuts [75,76,80], and two prospective studies in over 4000 older adults reported better overall cognition [78,79] and verbal memory [78].

To summarise evidence from observational studies, there was a consistent association between nut consumption and better cognitive function test scores, regardless of whether nut intake was investigated specifically, or when considered as part of an overall dietary pattern. For example, the adherence to healthy dietary patterns that included nuts (e.g., the Mediterranean or DASH diet) was associated with better cognitive function [75,79,80]. However, observational studies showed association, not causation. Participants’ health condition and long-term dietary habits prior to these studies may influence the study findings, and should also be considered.

### 4.2. Evidence from Interventional Studies

#### 4.2.1. Nut-Specific Studies

Three interventional studies that investigated the effects of nut supplementation on the cognitive function of older adults were identified (Table 4). Two more recent studies that had larger study sample sizes supplemented the intervention diets with almonds [81] or walnuts [82] as 15% of participants’ daily energy intake. Despite the larger dose and sample sizes, these studies did not identify significant differences in cognitive performance or mood after the intervention periods [81,82]. On the other hand, a pilot study that supplemented the diet with one Brazil nut (~5 g) per day for 6 months reported improvements in two (verbal fluency and constructional praxis) of the six subsets of the Consortium to Establish a Registry for Alzheimer’s Disease (CERAD) neuropsychological tests battery [83]. The reason for the contradicting findings from these interventional studies is unclear; the authors of the Brazil nut study attributed the findings to the antioxidative activities of selenium and glutathione peroxidase enzyme in the Brazil nut. It is also possible that the pilot study included participants with mild cognitive impairment, while the other two studies included healthy older adults; hence, they were more likely to detect a difference between the intervention and control groups. 

#### 4.2.2. Studies on Dietary Patterns That Include Nuts

Besides the nut-specific studies, three studies also incorporated nuts as part of an overall Mediterranean diet intervention [84,85,86]. These studies included large sample sizes and were conducted over a longer period of time, ranging from 6 months to 6.5 years. Consuming 30 g/day of mixed nuts as part of a Mediterranean diet was shown to improve memory composite scores (*p* = 0.04) but not frontal and global cognition when compared to a low-fat control diet [86]. Two other studies that also used a similar Mediterranean dietary pattern intervention approach also reported better cognitive function test scores after the interventions [84,85]. However, the differences between the intervention and the control group disappeared after the findings were adjusted for multiple factors including incident depression.

### 4.3. Summary

Most epidemiological studies (of both cross-sectional and prospective study designs) appear to show positive associations between nut consumption and the cognitive function of older adults. These epidemiological studies were conducted in countries from different regions (Asia Pacific, Europe, and North America). Not surprisingly, almost all observational studies had larger study populations than the interventional studies. 

Although one study reported a sex-specific association between nut intake and cognitive function (better in men) [80], other observational studies included in this review that recruited both sexes did not support this observation. In fact, two out of three observational studies that included only females in their studies [73,78] reported positive associations between nut intake and better cognitive scores. Therefore, based on the overall observations, the relationship between nuts and better cognitive performance is likely to be generalisable to all older men and women globally. However, more future studies are still needed to either confirm or rule out the sex-specific associations between nuts and cognition of older adults.

It should be noted that while positive associations between nut consumption and cognitive function in older adults were found in cross-sectional and prospective (with long follow-up) observational studies, almost all interventional studies failed to demonstrate the benefits of nut supplementation (alone or as part of an overall dietary pattern intervention) on cognitive function measurements. The inconsistent findings between studies of different designs suggest that the benefits of nuts on cognition may potentially require very long-term habitual nut consumption. Hence, the effects of nuts were not detected in interventional studies that were generally shorter in study intervention periods. Relatively smaller sample populations in the interventional studies may be another reason why statistically significant effects of nuts on cognitive function of older adults were not detected. The relatively small associations reported by observational studies with large sample sizes suggest that large interventional studies may be required in the future. Regardless, it is important to consider the clinically meaningful effect size too, so that emphasis is not placed on statistical power alone.

In the interventional studies that used an overall dietary pattern approach, nut consumption was only one component of the overall interventions, making it difficult to assess the independent effects of nuts, especially if the study sample populations were small due to the high burden of an interventional study design. In addition, almost all interventional studies included healthy community-dwelling older adults, which may have reduced the likelihood to detect further improvements in cognitive function within a short period of intervention. This speculation is supported by a pilot study that included older adults who had mild cognitive impairment, where significant improvement in cognitive function was found after a very low dose of nut supplementation for six months [83]. 

## 5. Conclusions and Future Directions

Overall, there are some preliminary data suggesting that nut consumption may be associated with longer telomere length, lower risk of sarcopenia, and better cognition in older adults. The associations appear to be more consistent when nuts were considered as part of the overall diets of older adults, suggesting a synergistic effect between nuts and other food groups. However, the evidence to-date is largely based on observational studies, and the findings were not always consistent. Future research is warranted to confirm these associations. This includes observational studies that are longer-term and adequately powered, because changes to function and cognition occur over time. Well-designed, long-term clinical studies are also needed to confirm the causal relationships between nuts and these health aspects of older adults, and whether the effects from nuts are clinically meaningful. Future research will be needed to form and guide the development of specific nut recommendation for older adults’ health.

## Figures and Tables

**Table 1:** Nut consumption and telomeres.

Author, Year	Study Design	Study Participants	Dietary Assessment Method	Dietary Patterns Assessed	Measure of Telomere	Outcomes
Boccardi, 2013 (Italy)	Cross-sectional	*n* = 217 elderly Caucasians (*n* = 115 men, *n* = 102 women); age range: 81–87 years, mean 78.0 ± 2.7 years	Dietary questionnaire	Mediterranean Diet Score (MDS) (Trichopoulou 2003)	Leukocyte telomere length and telomerase activity PBL/qPCR (Cawthon, 2002)	Greater adherence to MDS associated with longer LTL (*p* = 0.003) and higher telomerase activity (*p* = 0.013), and remained significant after adjustment. Every year increase in age LTL decreased by 0.072 Kb, 0.057 Kb, and 0.051 Kb in low, medium, and high adherence respectively (*p* = 0.001).
Crous-Bou, 2014 (USA)	Cross-sectional from the Nurses’ Health Study	*n* = 4676 females; age range: 42–70 years, mean 59 ± 6.6 years	Semi-quantitative FFQ	Alternative Mediterranean Diet Score (AMDS) (Trichopoulou 2003)	Leukocyte telomere length PBL/qPCR (Cawthon, 2002)	Greater adherence to the AMDS was associated with LTL *z*-score (*p* for trend = 0.02 (without adjustment); and *p* for trend = 0.004 after adjustment). No independent association with nut consumption (*p* for trend = 0.24).
Garcia-Calzon, 2016 (Spain)	Cross-sectional analysis of the PREDIMED-NAVARRA trial	*n* = 520 (*n* = 234 male, *n* = 286 female) at high risk of CVD; age range 60–80 years females, 55–80 years males; mean age 67 ± 6.0 years	FFQ and 14-item questionnaire to evaluate adherence to Mediterranean dietary pattern	Mediterranean diet adherence score (MedDiet)	Leukocyte telomere length PBL/qPCR (Cawthon, 2002)	Higher adherence to MedDiet was associated with greater age-adjusted *z*-score LTL) and a lower risk of having short telomeres in women, but not men. Nut intake was not associated with LTL.
Gu, 2015 (USA)	Cross-sectional	*n* = 1743, aged ≥65 years; *n* = 506 white, *n* = 536 African American, *n* = 679 Hispanic, *n* = 22 other	Semi-quantitative FFQ	Mediterranean Diet Score	Leukocyte telomere length PBL/qPCR (Cawthon, 2009)	No association overall between MDS and telomere length. There was a positive association among Whites, 1 unit increase in MDS corresponds to 48 bp increase in LTL, but not African Americans or Hispanics. No association between nuts and telomere length.
Leung, 2018 (USA)	Cross-sectional analysis of NHANES 1999–2002	*n* = 4758 (*n* = 2208 males, *n* = 2550 females), age range 20–75 years, mean age 39.5 years	Single 24 h recall	Healthy Eating Index 2010 scores (HEI-2010); Alternative Healthy Eating Index 2010 scores (AHEI-2010); Mediterranean Diet scores (MDS), Dietary Approaches to Stop Hypertension (DASH) score	LTL from whole blood (Cawthon, 2002)	Comparison of the top and bottom quintiles showed higher scores for all diet quality indices were associated with longer telomere length in women, but not men.
Meinilä, 2019 (Finland)	Cross-sectional and prospective (mean follow up period 9.9 y for females and 9.7 y for males)	*n* = 1046 (*n* = 456 men, *n* = 590 women); mean age 61 years	Semi-quantitative FFQ	Baltic Sea diet score (BSDS); modified Mediterranean diet score (mMed); Dietary Inflammation index (DII)	Leukocyte telomere length PBL/qPCR (Cawthon, 2009)	Adherence to the any of the 3 dietary indices was not associated with LTL in the cross-sectional analysis. In the prospective analysis adherence to mMed was associated with slightly higher rates of shortening in women (largely driven by the fruit and nut food component)—this was not considered clinically important.
Milte, 2018 (Australia)	Cross-sectional	*n* = 679 (*n* = 330 men, *n* = 349), age range 55–65 years, mean age 62.7 years	FFQ	Dietary Guideline Index; Recommended Food Score; Mediterranean Diet Score (MDS)	LTL from whole blood (Cawthon, 2002)	There were no associations between any of the diet indices and LTL.
Ojeda-Rodriguez, 2019 (Spain)	Cross-sectional (SUN Project)	*n* = 886 (*n* = 645 males, *n* = 241 females), aged ≥55 years	Semi-quantitative FFQ	Prime Diet Quality Score (PDQS); Fat Quality Index (FQI); Alternative Healthy Eating Index 2010 scores (AHEI-2010); Mediterranean Diet Adherence Screener (MEDAS), Dietary Approaches to Stop Hypertension (DASH)	Salivary telomere length PBL/qPCR (Cawthon, 2009)	There were fewer participants with short telomeres in the top tertile for each diet quality index, specifically the PDQS, MEDAS and DASH in crude and adjusted models; and all indices for adjusted models.
Ventura Marra, 2019 (USA)	Cross-sectional	*n* = 96 (*n* = 41 men, *n* = 55 women), aged 45–60 years, with at least one risk factor for CVD	Three 24 h recalls for the HEI-2015 index and aMed; 24-item questionnaire for the DST	Healthy Eating Index 2015 (HEI-2015); alternative Mediterranean diet score (aMed); Dietary Screening Tool (DST)	LTL from whole blood (Cawthon, 2002)	There were no associations between the HEI-2015 or aMed and LTL. Those scoring “at risk” by the DST were more likely to have short LTL.
Chan, 2010 (Hong Kong)	Cross-sectional	*n* = 2006 (*n* = 976 men, *n* = 1030 females), aged ≥65 years	FFQ	Included food group nuts/legumes/seeds	LTL from whole blood (Cawthon, 2002) with modifications	No association between the nuts/legumes/seeds group and LTL.
Gong, 2017 (China)	Cross-sectional	*n* = 553 (*n* = 281 men, *n* = 272 women); mean age: 45.1 years men; 48.3 years women	FFQ	PCA was used to derive 4 dietary patterns: Vegetable-rich (higher in fruits, vegetables, whole grains, dairy products, nuts, eggs, tea); Macho; Traditional; High energy-density	Leukocyte telomere length (Zhao, 2009)	Only the Vegetable-rich dietary pattern was associated with longer TL in women, but not men. The longer length of 160 bp corresponded with a difference of 4 years of aging
Karimi, 2018 (Iran)	Cross-sectional	*n* = 300 men, aged 25–40 years	Semi-quantitative FFQ	PCA was used to derive 4 dietary patterns: healthy diet pattern, Western pattern, traditional pattern, vegetarian diet pattern. Nuts and seeds were examined as a food group	LTL from whole blood (Cawthon, 2002)	Nuts and seeds were negatively, but not statistically significantly, associated with LTL.
Lee, 2015 (Korea)	Prospective (10 y follow up)	*n* = 1958 (*n* = 1018 men, *n* = 940 women), age range 40–69 years baseline	Semi-quantitative FFQ	Factor analysis characterised a Prudent dietary pattern and a Western dietary pattern	LTL from whole blood (Cawthon, 2002)	The Prudent dietary pattern was positively associated with LTL. The Western diet was not associated with LTL. Nuts were one food component positively associated with LTL.
Nettleton, 2008 (USA)	Cross-sectional of the Multi-Ethnic Study of Atherosclerosis (MESA) study	*n* = 840 (*n* = 434 women, *n* = 406 men), *n* = 157 whites, *n* = 228 African Americans, *n* = 455 Hispanics), age range 45–84 years	Semi-quantitative FFQ	PCA was used to derive 2 dietary patterns: fats and processed meat; and whole grains and fruits (including nuts). Nuts or seeds were also analysed as a food group component.	LTL from whole blood (Cawthon, 2002)	No association between dietary pattern andLTL. The food group nuts or seeds was not associated with LTL.
Zhou, 2016 (China)	Cross-sectional	*n* = 556 (*n* = 213 males, *n* = 343 females), mean age early 50s	Semi-quantitative FFQ	Nuts and seeds food group was examined	Leukocyte telomere length PBL/qPCR (Cawthon 2009)	Intakes of nuts or seeds were highest among those in the upper tertile for telomere length. Intakes of nuts and seeds were positively associated with telomere length.

Abbreviations: AHEI-2010, Alternative Healthy Eating Index; AMDS, Alternative Mediterranean Diet Score; bp, base pairs; CVD, cardiovascular disease; DASH, Dietary Approaches to Stop Hypertension; DII, DIetary Inflammatry Index; FQI, Fat Quality Index; LTL, leukocyte telomere length; MDS, Mediterranean Diet Score; MEDAS, Mediterranean Diet Adherence Screener; *n*, number; NHANES, National Health and Nutrition Examination Survey; PCA, principal component analysis; PDQS, Prime Diet Quality Score; PREDIMED, Prevención con Dieta Mediterránea trial; SUN, Seguimiento Universidad de Navarra.

**Table 2:** Studies on nuts and sarcopenia related factors.

Author (Year) Study Location	Study Design	Study Participants	Dietary Assessment Method	Nuts/Dietary Patterns Assessed	Measure of Functional or Related Outcomes	Results
**Nut-specific studies**						
Arias-Fernández, 2019 (Spain)	Prospective study: Seniors-ENRICA cohort Cohort was established in 2008–2010, with 7.2 years of follow-up	3289 individuals aged ≥60 years	A validated computerised diet history was used to assess nut consumption in 2008–2010 and 2012. Average nut consumption at baseline (2008–2010) and in the first follow-up wave of data collection (2012) was calculated to represent cumulative intake over follow-up.	Diet history included 20 types of nuts, which were grouped as follows: almonds, hazelnuts, peanuts, chestnuts, walnuts, pine nuts, sunflower seeds, pistachios, sesame seeds, cashews, macadamia nuts, and other types of nuts.	Five domains were considered to characterise participants’ physical function: (1) Agility: Rosow and Breslau scale (self-reported) (*n* = 1502) (2) Mobility: Rosow and Breslau scale (self-reported) (*n* = 1502) (3) Overall physical function: physical component summary (PCS) score of the 12-Item Short-Form Health Survey SF-12 (self-reported) (*n* = 1665) (4) Grip strength: highest value in two consecutive measures on the dominant hand (objective measure of muscle strength) (*n* = 1256) (5) Gait speed: 3 m walking speed test (objective measure of physical performance) (*n* = 1233)	In men, compared with no consumption, an intake of nuts ≥11.5 g/d (median) in nut consumers was associated with lower risk of self-reported impaired agility (fully-adjusted HR = 0.59, 95% CI: 0.39–0.90) and mobility (fully-adjusted HR = 0.50, 95% CI: 0.28–0.90). In women, compared with no consumption, the fully-adjusted HR (95% CI) of impaired self-reported overall physical function was 0.65 (0.48–0.87) for intake ≥11.5 g/d. No association was found between nut consumption and grip strength and gait speed.
**Studies on dietary patterns that include nuts (as a food group)**						
Bradlee, 2018 (United States)	Prospective study: Framingham Offspring Study Began in 1972, with a median follow-up of 13.0 years	5124 offspring were enrolled in 1972 For skeletal muscle mass outcomes, participants aged 40 years or older were included. For functional status outcomes, participants aged 50 years or older at the time of the dietary assessments were included; follow-up for functional status outcomes continued for up to 16 years.	Diet records (six days)	Protein-source foods: Legumes, Soy, Nuts, Seeds	Skeletal muscle mass was estimated using BIA. Functional status was measured using standardised instruments: (1) Rosow–Breslau scale measures gross-mobility capacity (2) Nagi scale assesses self-reported functional limitations	Higher intake of “legumes, soy, nuts and seeds” was associated with higher percent skeletal muscle mass over 9 years. In men, compared with consumption <0.25 serving/day of legumes, soy, nuts and seeds, those who consumed ≥1.25 serving/day had higher percent skeletal muscle mass (36.8% vs. 37.5%, *p* = 0.0197). In women, compared with consumption <0.25 serving/day of legumes, soy, nuts and seeds (27.3%), those who consumed 0.25 to <1.25 serving/day and ≥1.25 serving/day had higher percent skeletal muscle mass (28.2% and 28.1% respectively, both *p* ≤ 0.0156). In the multivariable Cox proportional hazards models, “legumes, soy, nuts and seeds” was not a predictor of limitation in two or more functional tasks from the Rosow–Breslau and Nagi scales (HR = 0.96, 95% CI: 0.72, 1.30).
Hai, 2017 (China)	Cross-sectional study	848 individuals aged ≥60 years who lived in the community for more than 12 months. Data from 834 participants were used for the analysis.	A validated simplified FFQ was used. Frequency units: day, week, month or never.	Nine food categories based on the Chinese Food Guide Pagoda: (1) Grain or cereals (2) Vegetables (3) Fruit (4) Meat (pork, beef, poultry, and mutton) (5) Eggs (6) Fish and shrimp (7) Milk and milk products (8) Legumes (9) Nuts	Sarcopenia, i.e., presence of low muscle mass, plus low muscle strength or low physical performance. Muscle mass was measured using BIA. Grip strength was measured using a dynamometer. Usual gait speed (m/s) on a 6 m course was used to measure physical performance and a slow walking speed was defined as a walking speed <0.8 m/s.	In females, participants with sarcopenia had significantly lower frequency of nut consumption than those without sarcopenia (0.05 times vs. 0.81 times per week, *p* = 0.022). This was not found in male participants (*p* = 0.135). After adjusting for potential confounders, there was a significant association between prevalence of sarcopenia and frequency of nut consumption per week (OR = 0.724, 95% CI: 0.532, 0.985, *p* < 0.05).
Lim, 2020 (Korea)	Cross-sectional study. 2008 to 2011 Korea National Health and Nutrition Examination Survey (KNHANES).	3350 elderly over 65 years, 862 had sarcopenia.	24 h dietary intake	Food intake analysis was based on the guideline of 15 food groups: (1) Cereals (2) Potato and starches (3) Sugars and sweeteners (4) Pulses (5) Nuts and seeds (6) Vegetables (7) Fungi and mushrooms (8) Fruits (9) Meat (10) Eggs (11) Fish and shellfish (12) Seaweeds (13) Milk (14) Oil and fat (15) Beverages	Sarcopenia was defined as muscle mass excluding bones and fats of limbs measured by dual energy X-ray absorptiometry divided by weight in the form of percent is under the twice of standard deviation.	In males, the sarcopenia group had significantly lower intake of nuts and seeds than the non-sarcopenia group (5.2 g/day vs. 3.1 g/day, *p* = 0.002). This was not found in female participants (*p* = 0.258). Logistic regression analyses showed no significant association between prevalence of sarcopenia and tertiles of nut and seed intake in both males and females.
**Studies on dietary patterns that include nuts (diet quality indices)**						
Ballesteros, 2020 (Spain)	Prospective study: Seniors-ENRICA cohort Cohort was established in 2008–2010, with a median follow-up of 3.5 years	3289 individuals aged ≥60 years 2071 included in the analysis	A validated computer-assisted face-to-face dietary history.	Mediterranean Diet Adherence Screener (MEDAS) score was used to determine the adherence to the Mediterranean diet, with a higher score indicating greater adherence.	Risk of falling	There was an inverse dose-response relationship between the MEDAS score and the risk of falling in older adults (*p* for trend = 0.04). Compared with the people in the lowest tertile of the MEDAS score, those in the second tertile (OR = 0.93, 95% CI: 0.71–1.21) and highest tertile (OR = 0.72, 95% CI: 0.53–0.98) showed lower risk of falling after adjustment for potential confounders.
Schacht, 2019 (Denmark)	Cross-sectional study	184 Danish older individuals aged 65 years and above participated in the “Counteracting Age-related Loss of Skeletal Muscle Mass” (CALM) study.	3 days weighed food diaries from Wednesday to Friday. Average daily consumption of different food products was calculated.	Dietary index characterised by higher intakes of whole grains, dairy products, fish, legumes, nuts, fruit, and vegetables.	Muscle function (1) 30s chair stands (2) 400 m gait speed (3) Handgrip strength (dynamometer DHD-1 [SH100]) (4) Knee extensor maximal voluntary contractions was measured using an isokinetic dynamometer	Dietary index was associated with faster 400 m walking speed (*p* for trend = 0.021). No associations were found between dietary index and 30s chair stands, handgrip strength, knee extensor maximal voluntary contractions (all *p* for trend > 0.05).
Hashemi, 2015 (Iran)	Cross-sectional study	300 elderly men and women aged 55 years and older	Semi-quantitative Food Frequency Questionnaire, frequency of 117 common Iranian food items by standard serving size	Mediterranean pattern was defined as a dietary pattern with high factor loadings (>0.4) in food groups such as olives and olive oil, low and high carotenoid vegetables, tomatoes, whole grains, nuts, fish, fresh and dried fruits, and pickles.	Sarcopenia is defined as low appendicular muscle mass with either low muscle strength or low muscle performance. Muscle mass (DXA) was calculated as the ratio of total lean mass of legs and arms (ASM) to squared height. Muscle strength was measured using a handgrip dynamometer. Muscle performance was measured using a 4 m walk gait speed test. Low muscle performance was defined as gait speed <0.8 m/s.	There was a significant association between Mediterranean dietary pattern and prevalence of low gait speed (*p* = 0.02). The percentage of participants with low gait speed (< 0.8 m/s) in the top tertile was 29.3%, second tertile was 47.5%, and lowest tertile was 43.9%. After adjusting for potential confounders, Mediterranean diet was associated with lower odds of having sarcopenia. Odds ratio (95% CI): T1: 1.00 T2: 0.84 (0.40–1.70) T3: 0.40 (0.17–0.97) *P* for trend: 0.04

**Table 3:** Observational studies examining the association between nut consumption and cognition.

Author	Participants	Controls	Comparators	Nut Type	Design	Cognitive Test	Results
**Nut-specific studies**							
Arab, 2015 (USA)	*n* = 5054 (NHANES III) and *n* = 2975 (NHANES 1999–2002) adults aged 60 years and over	No nut intake	Tertiles of intake of walnuts with other nuts (WWON), or walnuts with high certainty (WWHC).	Walnuts and other nuts	Cross-sectional, observational	NHANES III included the Story Recall Test (SRT) as a test of cognitive attention and delayed verbal memory. NHANES 1999–2002 included the Digit Symbol Substitution Test (DSST) that assessed response speed, sustained attention, visual spatial skills and associative learning and memory	Significantly higher SRT and DSST in the WWHC group than non-consumers after adjusting for covariates such as age, gender, race, education, BMI, smoking, alcohol, and physical activity.
Nurk, 2010 (Norway)	*n* = 2031, aged 70–74 years	Low intake or non-consumers of nuts	High intake of nuts	All nut types	Cross-sectional, observational	The cognitive test battery included Kendrick Object Learning Test, Trail Making Test—part A, modified versions of the Digit Symbol Test, Block Design, Mini-Mental State Examination and Controlled Oral Word Association Test	No significant differences in all cognitive tests after adjusting for multiple factors (sex, education, suppl use, smoking, metabolic disease, intake of dairy, meat, fish, fat, protein)
Valls-Predet, 2012 (Spain)	*n* = 447, 52% women, aged 55–80 years (mean 66.9 years), adults with high cardiovascular risk	Low nut intake	High nut intake	Walnuts, per 30 g/d increase	Cross-sectional, observational	The instruments included MMSE, Rey auditory verbal learning test (RAVLT) for immediate and delayed memory, verbal paired associates test from Wechsler Memory Scale (WMS), semantic verbal fluency test, digit span test of the Wechsler Adult Intelligence Scale (WAIS), and Color Trail Test	Significant associations between walnuts with working memory [Regression coefficient, B = 1.191 (0.061–2.322)] (*p* = 0.039)
Li, 2019 (China)	*n* = 4822, aged 62–67 years from China Health and Nutrition Survey 1991–2006	Non-consumers of nuts, 0.1–9.9 g/d	Consumed nuts >10 g/d	All nut types	Prospective observational, 15-year follow-up	Modified Telephone Interview for Cognitive Status. Poor cognitive function was defined as cognition score < 7.	The unadjusted cognitive score decreased by 0.29 (95% CI 0.22–0.28) with every one-year aging during 1997–2006. Nut intake of more than 10 g/d was associated with higher cognition score by 0.63 points (95% CI 0.15–1.12) or 40% less likely to have poor cognitive function (OR 0.60, 95% CI 0.43–0.84) after adjusted for demographic, lifestyle behaviour, BMI, energy intake
O’Brien 2014 (USA)	*n* = 15467, women age ≥70 years (mean 74 years)	Non-consumers of nuts	Higher intake of nuts	All nut types	Prospective observational, 15–20-year follow-up	The telephone interview for Cognitive status (TICS), a global score averaging the results of all tests (TICS, immediate and delayed verbal recall, category fluency, and attention), and a verbal memory score averaging the results of tests of verbal recall.	Higher long-term total nut intake was associated with better average cognitive status for all cognitive outcomes. Positive associations between nut intake and 10-word list immediate recall (*p*-trend = 0.0015), and digit span backwards test (*p*-trend = 0.01) Women consuming at least 5 servings of nuts/week had higher global composite scores than non-consumers (mean difference = 0.08 standard units, 95% confidence interval 0.00–0.15; *p*-trend = 0.003). Nut intake was not significantly associated with rate of cognitive decline on any of the tests
Rabassa, 2019 (Italy)	*n* = 119, aged 73 ± 6 years	Non-consumers of nuts (*n* = 72)	Nut consumers (>2.9 g/d) (*n* = 47)	All nut types	Prospective observational, 3-year follow-up	Mental State Examination (MMSE) administered at baseline and at 3 y follow-up	Nut consumption estimated either by the dietary marker or by the urinary marker model is in both cases associated with less cognitive decline (OR: 0.78, 95% CI: 0.61,0.99; *p* = 0.043 and OR: 0.995, 95% CI: 0.991,0.999; *p* = 0.016, respectively)
**Studies on dietary patterns that include nuts**							
De Amicis, 2018 (Italy)	*n* = 279, 80 men, 199 women, aged 67–74 years	Habitual diet (*n* = 195)	Adherence to MedDiet (*n* = 84)	All nut types, per 30 g/d increase	Cross-sectional, observational	Mini-Mental State Examination (MMSE). An MMSE ≥ 24 = normal cognitive function; MMSE 20–23 = suspected cognitive impairment; MMSE ≤ 19 = mild cognitive impairment. Age and education correction applied	MedDiet was associated with a lower risk of cognitive impairment (odds ratio [OR] D 0.39; 95% confidence interval [CI], 0.15–0.99; *p* = 0.045), as was the consumption of wine (OR D 0.37; 95% CI, 0.16–0.84; *p* = 0.018) and nuts (OR = 0.30; 95% CI, 0.13–0.69, *p* = 0.005).
Dong, 2015 (China)	*n* = 894, aged 55–76 y, Chinese adults without Alzheimer’s or Parkinson’s Disease	Healthy adults (*n* = 646)	Adults with mild cognitive impairment (*n* = 248)	All nut types	Cross-sectional, observational	Montreal Cognitive Assessment (MoCA) test	The nut intake of MCI patients (15.35 g/d) was less than the healthy subjects (17.12 g) (*p* < 0.05). No associations with MOCA scores
Katsiardanis, 2013 (Greece)	*n* = 557, 237 men and 320 women aged 65 years and over	Adults with cognitive impairment (MMSE < 24) (*n* = 331)	Healthy adults (MMSE > 24) (*n* = 226)	All nut types	Cross-sectional, observational	Mini-Mental State Examination (MMSE) assessments	Adherence to the Mediterranean diet was positively associated with MMSE score in men (*p* = 0.02), but inversely associated in women (*p* = 0.04). Intake of pulses, nuts, and seeds was associated with lower likelihood of having MMSE score < 24 in men (*P* = 0.003). Individual nutrients did not achieve significance (7.4 vs. 11.0 g/d)
Samieri, 2013 (USA) (a)	*n* = 6174, women aged >65 years (mean 72 ± 4 years) from the Women’s Study	Alternate MedDiet scores and lowest quintile of nut intake	Alternate MedDiet scores and highest quintile of nut intake	All nut types	Prospective observational, 9-year follow-up	The Telephone Interview for Cognitive Status (an adaptation of MMSE), East Boston Memory Test (immediate and delayed recalls), Telephone Interview for Cognitive Status ten-word list, category fluency. Two primary outcomes were composite scores of global cognition and verbal memory.	Alternate Mediterranean diet score was not associated with trajectories of repeated cognitive scores (*p* for score quintiles median × time interaction = 0.26 for global cognition and 0.40 for verbal memory), nor with overall global cognition and verbal memory at older ages, assessed by averaging the three cognitive measures (*p* trend = 0.63 and 0.44, respectively). No significant trend in nut intake and global cognition and verbal memory.
Samieri, 2013 (USA) (b)	*n* = 6174, women aged ≥70 years (mean 74 years) from the Nurses’ Health Study	Low MedDiet adherence and low nut intake	High MedDiet adherence and high nut intake	All nut types	Prospective observational, 6 years follow-up	Telephone Interview for Cognitive Status (TICS) and composite scores of verbal memory and global cognition	MedDiet was not associated with decline in global cognition or verbal memory. Each higher quintile of long-term MedDiet score was linearly associated with better multivariable-adjusted mean cognitive scores [differences in mean Z-scores between extreme quintiles of MedDiet = 0.06 (95% CI: 0.01, 0.11); = 0.05 (95% CI: 0.01, 0.08); and = 0.06 (95% CI: 0.03, 0.10) standard units; *p*-trends = 0.004, 0.002, and <0.001 for TICS, global cognition, and verbal memory, respectively]. Greater intake of nuts was associated with higher mean cognitive function in later life: global score *p* = 0.02, verbal memory *p* = 0.05)
Wengreen, 2013 (USA)	*n* = 3831, men and aged 65 years and over	Low adherence to DASH/ MedDiet and intake of nuts and legumes	High adherence to DASH/ MedDiet and intake of nuts and legumes	All nut types, per serving/d increase	Prospective observational, 11 years follow-up	Modified Mini-Mental State Examination (3MS), tested ≤4 times over 11 y	Higher DASH and Mediterranean diet scores were associated with higher average 3MS scores. People in quintile 5 of DASH averaged 0.97 points higher than those in quintile 1 (*p* = 0.001). The corresponding difference for Mediterranean quintiles was 0.94 (*p* = 0.001). These differences were consistent over 11 years. Higher intakes of whole grains and nuts and legumes were also associated with higher average 3MS scores [mean quintile 5 compared with 1 difference: 1.19 (*p* < 0.001), 1.22 (*p* < 0.001), respectively].

**Table 4:** Interventional studies examining the effects of nut consumption on cognition.

Author	Participants	Controls	Comparators	Nut Type	Design	Cognitive Test	Results
**Nut-specific interventional studies**							
Cardoso, 2016 (Brazil)	*n* = 20, 6 males 14 females, older adults (78 ± 5 years) with mild cognitive impairment	No nuts (*n* = 9)	Nut/ selenium supplementation (*n* = 11)	Brazil nut, 1 nut/day (288.75 ug/d Selenium)	Interventional, 6 months	CERAD neuropsychological battery	Improvements in verbal fluency (*p* = 0.007) and constructional praxis (*p* = 0.031) in nuts group
Coates, 2020 (Australia)	*n* = 128, aged 50–80 years (65 ± 8 years) who were overweight or obese	Nut-free diet (*n* = 65)	Almond enriched diet (*n* = 63)	Almonds providing 15%E	Interventional, 12 weeks	Computerised Mental Performance Assessment System (COMPAS) neuropsychological test battery.	No significant changes in cognitive performance or mood
Sala-Vila, 2020 (USA and Spain)	*n* = 708 free-living older adults, 68% women, aged 63–79 years, *n* = 636 completed the study	No nuts	Walnuts	Walnuts providing 15%E	Interventional, 2 years	A comprehensive neurocognitive test battery, calculated as the global cognition composite score	Modified intention-to-treat analysis did not find significant differences in global cognition composite scores between groups in the entire sample.
**Interventional studies on dietary patterns that include nuts**							
Knight, 2016 (Australia)	*n* = 152, healthy men and women aged 65 years and above, with normal cognitive function	Habitual diet (*n* = 72)	Adherence to MedDiet (i.e., vegetables, fruits, olive oil, legumes, fish, whole grain cereals, nuts and seeds, and low consumption of processed foods, dairy products, red meat and vegetable oils) (*n* = 80)	Walnuts, peanuts and almonds	Interventional, 6 months	Non-pathological changes in aspects of fluid cognition measured as executive function, memory, speed of processing, and visual-spatial memory ability	The MedDiet group did not perform better than the Habitual Diet control group for executive functioning (adjusted mean differences: +2.53, 95% CI -2.59 to 7.65, *p* = 0.33); speed of processing (adjusted mean differences: +3.24, 95% CI -1.21 to 7.70, *p* = 0.15); memory (adjusted mean differences: +2.00, 95% CI -3.88 to 7.88, *p* = 0.50); visual-spatial ability (adjusted mean differences: +0.21, 95% CI -0.38 to 0.81, 0.48); and overall age-related cognitive performance (adjusted mean differences: +7.99, 95% CI -4.00 to 19.9, *p* = 0.19)
Martínez-Lapiscina, 2013 (Spain)	*n* = 522, 44.6% men, aged 75 ± 6 years	Low-fat control diet	MedDiet with EVOO or mixed nuts	Walnuts (15 g), almonds (7.5 g), hazelnuts (7.5 g)	Interventional, 6.5 years	Mini-Mental State Examination (MMSE) and Clock Drawing Test (CDT) after 6.5 years of nutritional intervention	The adjusted means of MMSE and CDT scores were higher for participants in the MedDiet + Nuts than control (adjusted differences: +0.57 (95% CI +0.11 to +1.03), *p* = 0.015 for MMSE and +0.33 (95% CI +0.003 to +0.67), *p* = 0.048 for CDT). These results did not differ after controlling for incident depression.
Valls-Predet 2015 (Spain)	*n* = 334, 52.1% women, mean age 67 years, cognitively healthy adults	Low fat diet	MedDiet with extra virgin olive oil (EVOO) or mixed nuts	Mixed nuts (30 g/d)	Interventional, median 4.1 years (1.0–8.8 years)	Neuropsychological test battery: Mini-Mental State Examination, Rey Auditory Verbal Learning Test (RAVLT), Animals Semantic Fluency, Digit Span subtest from the Wechsler Adult Intelligence Scale, Verbal Paired Associates from the Wechsler Memory Scale, and the Color Trail Test. The z scores of change in each test to construct 3 cognitive composites: memory, frontal (attention and executive function), and global.	Adjusted cognitive composites (mean z-scores with 95% CIs) for changes above baseline of the memory composite were 0.04 (−0.09 to 0.18) for the MedDiet + EVOO, 0.09 (−0.05 to 0.23; *p* = 0.04 vs controls) for the MedDiet + nuts, and −0.17 (−0.32 to −0.01) for the control diet. Changes from baseline of the frontal cognition composite were 0.23 (0.03 to 0.43; *p* = 0.003) for MedDiet + EVOO, 0.03 (−0.25 to 0.31) for MedDiet + nuts, and −0.33 (−0.57 to −0.09) for control. Changes from baseline of the global cognition composite were 0.05 (−0.11 to 0.21, *p* = 0.005) for the MedDiet + EVOO, −0.05 (−0.27 to 0.18) for the MedDiet + nuts, and −0.38 (−0.57 to −0.18) for control.

CERAD, Consortium to Establish a Registry for Alzheimer’s Disease; CDT, Clock Drawing Test; COMPAS, Computerised Mental Performance Assessment System; DASH, Dietary Approaches to Stop Hypertension; DSST, Digit Symbol Substitution Test; EVOO, extra virgin olive oil; MedDiet, Mediterranean diet; MMSE, Mini Mental State Examination; MoCA, Montreal Cognitive Assessment; NHANES, National Health and Nutrition Examination Survey; RAVLT, Rey Auditory Verbal Learning Test; SRT, Story Recall Test; TICS, Telephone Interview for Cognitive Status; WAIS, Wechsler Adult Intelligence Scale; WMS, Wechsler Memory Scale; WWON, walnut with other nuts; WWHC, walnuts with high certainty.

## Data Availability

No new data were created or analyzed in this study. Data sharing is not applicable to this article.
